# What Shall We Do with Our Insane?

**Published:** 1842-02

**Authors:** Edward Jarvis

**Affiliations:** Louisville, Kentucky


### WESTERN JOURNAL

OF

### MEDICINE AND SURGERY.

FEBRUARY, 1842.

### Art. I.—*What shall we do with our Insane?* By Edward Jarvis, M. D., of Louisville, Kentucky.

What shall we do with our Insane? This is a question that must come home to all of us, whether physicians or citizens, and sometimes appeal with fearful earnestness to our hearts and understandings for answer. And are we ready to give it? Every one of us is liable to find insanity, in some form or other, in his own family. Every physician may be called, at any time, to see it among his friends. His advice is then pressingly asked—What shall be done with the patient suffering from moral or intellectual derangement?

This is a question—not, indeed, of life or death—for insanity is not a dangerous disease; comparatively few need die of it. But it is a question of future soundness of reason and of correct feelings, or of permanent insanity. And whether our patients or our friends shall be brought back to mental life and all its enjoyments, to usefulness, society, and to happiness; or whether the incubus of lunacy shall weigh forever upon their understandings, their feelings and affections be enchained or perverted, and they become a burden upon their families, a terror to society, or perhaps, sinking into fatuity, a mockery to the unthinking and the heartless; which of these shall happen, depends upon us, and our answer to the question—What shall be done with the insane?

Nor is this a rare question. It has been asked, or it ought to be asked, frequently; for there were, at the last national census, taken in the year 1840, eight hundred and thirty-three lunatics and idiots in Kentucky; eight hundred and fifty-one in Tennesee; five hundred and sixty-eight in Indiana; two hundred and twenty-seven in Illinois; two hundred and seventy-eight in Missouri, and thirteen hundred and sixty in Ohio.* And their claims press, with an irresistible importunity, upon our professional skill and upon our humanity, to tell them what they shall do. And though our fathers and we, hitherto, have not told them, and these insane patients have got no relief, and a large portion of them have become

* Statement of the number of Insane in the Western states, taken from the last national census, and how supported.

| | *Whites.* | | *Colored.* | | | | |
|---|---|---|---|---|---|---|---|
| | Public | Private | Private | Public | Total | Populat'n | Proportion of insane to population |
| Missisippi | 14 | 102 | 66 | 16 | 198 | 375,651 | 1 in 1,892 |
| Louisiana | 8 | 45 | 37 | 8 | 98 | 351,176 | 1 " 3,592 |
| Tennesee | 103 | 596 | 124 | 28 | 851 | 829,210 | 1 " 974 |
| Kentucky | 276 | 406 | 110 | 41 | 833 | 777,397 | 1 " 934 |
| Ohio | 363 | 832 | 62 | 103 | 1360 | 1,519,467 | 1 " 1,117 |
| Indiana | 110 | 383 | 46 | 29 | 568 | 683,314 | 1 " 1,203 |
| Illinois | 25 | 162 | 32 | 8 | 227 | 474,404 | 1 " 2,089 |
| Missouri | 44 | 165 | 52 | 17 | 278 | 381,102 | 1 " 1,370 |
| Arkansas | 3 | 14 | 6 | 5 | 28 | 95,642 | 1 " 3,415 |
| | 946 | 2705 | 535 | 255 | 4441 | 5,487,363 | 1 " 1,235 |

The average for all the United States is one to 990.

incurable maniacs, or have sunk into hopeless imbecility, yet we can no longer be silent; we must tell, what shall be done with the four thousand four hundred and forty-one lunatics and idiots, that live in the vallies of the Ohio and the Mississippi. The present state of medical science will not suffer these any longer to be neglected, and still less will it permit, that those, who hereafter shall become insane, shall have their disease permanently fixed upon them.

What then can be done? What can we do for them? As regards the future, these patients may well be divided into the hopeless and hopeful. In the former class are included idiots from birth, and, also, such as have become idiotic in the progress of the disease, epileptics, and paralytics. But although these may not hope for restoration to health, yet most of them may be improved and rendered more comfortable to themselves, and tolerable in society.

To the other class belong all other cases—old and recent. For these, there are three grades of hope; and according to the results of the treatment of these must they ultimately be classed, though not now.

1st. Those who may be completely restored to health.

2d. Those, who, though they may never enjoy perfect sanity, yet may be so far improved that they can live peaceably and quietly among their friends, without interfering with the safety or comfort of society.

3d. Those who can obtain neither of the former conditions, but may, under the influence and restraints of a hospital government, have their delusions so far modified, and their agitated feelings so soothed, that they may enjoy a calm and contented existence in an asylum, though they could not bear the irritations of the world, without distress or raving madness.

Before the insane fall into either of these classes, they must aim at the first; and if they fail there, they fall successively into the second, and even the third class, by the trial of the remedial measures. Few indeed are the lunatics, who may not be brought into one or the other of these classes; and if taken very early in the disease, and proper measures be faithfully used, few will be found who may not be put in the first class, as we shall hereafter show.

Our first object is to cure the lunatic. If this fails, our second object is to improve him, so that he may live comfortably at home. And lastly, in default of both these, our third object is, to find a comfortable resting place for him. How shall we obtain these?

Shall we retain the insane at their homes, and apply such remedies as we have, and such restraints as their excitements and our fears seem to demand? We have been trying this plan for many years in Kentucky and the other Western states, and have found, in most cases, no better result than permanent insanity, sometimes uncontrollable frenzy, and even dangerous madness, and sometimes irretrievable imbecility. And for all purposes of restoration to health, of improvement or comfort for the maniac, of satisfaction to friends, or even of public or private economy, the old system of keeping lunatics at their homes, in charge of committees or of their families, wandering freely abroad or confined in strong places, has been demonstrated to be worthless, in almost every town and neighborhood, and, without doubt, within the observation of every physician.

The great number of old cases, that were once recent, but have been suffered to become chronic, and probably permanent; the many imbecile that were once rational, and the uncontrollably excited maniacs, are melancholy proofs of how vain were our hopes that the disease might wear itself away, and how futile were all our attempts to cure it with any means and appliances of a domestic nature.

We must, then, try some other plan. Home is not the place for the cure of the insane. The circumstances and associations of home too often aggravate and perpetuate the mental disorder, rather than soothe and relieve it. So that now all medical authorities advise the removal of the patient from the familiar scenes and associates, among which the disorder first arose and grew.

Esquirol advises, that the insane be removed from their homes, and confined, for the five following reasons:

1. For their own security, and that of their families, and for the maintenance of public order.

2. To remove them from the influence of external circumstances, which may have produced their disorder, and may be likely to protract it.

3. To overcome their resistance to curative means.

4. To subject them to a regimen appropriate to their situations.

5. To cause them to resume their intellectual habits.

The first of these reasons is sufficiently obvious, and need not be urged here. The public and the friends of the lunatics are careful enough of their own safety and of the security of the latter, to place them out of harm's way, and beyond the means of doing mischief.

A large portion of the cases of lunacy arise directly from the trials, difficulties, and misunderstandings of home; from domestic grief, from fear of poverty, and from disappointments. Some are caused by excessive labor of body or mind, from hard study or intense application to business, and from unrequited love or thwarted ambition.

All these have their origin in matters and circumstances immediately about their usual abode and haunts. These causes, which at first excited, next continue to feed the disorder. In order then to cure the disease, we must first remove the cause from the patient, or the patient from the cause. And if that cause be home or its concomitants, removal to another place of residence must be the first step toward relief.

Most cases of lunacy are aggravated by the usual and familiar scenes and persons, whatever may have been the original cause of the derangement. The insane are generally suspicious of others, or self-distrustful. The ordinary motives cease to influence their opinions or govern their actions. The usual reasons do not draw their confidence. One conceives that he cannot perform the ordinary duties, which he has hitherto done cheerfully, and nothing will induce him to attempt them ; and he then is in great distress, because he has thus neglected what he knows he ought to do. Another supposes that his family are his enemies, and are seeking to injure him. Another is peculiarly irritable upon the common topics and interests of his neighborhood ; he imagines that they implicate him in wrong or danger, and flies in a passion when they are mentioned, or avoids allusion to them altogether. One has strange conceptions of his relations to his friends and the world ; he supposes himself to be a king, and therefore demands implicit obedience ; or a prophet, and expects unreserved faith in his opinions. To resist this assumed authority, to refuse this undue submission, or to withhold this groundless belief, would but aggravate the lunatic's derangement of mind, or exasperate the rage of the maniac, and is impossible for the family and friends, without increasing the disorder. At home, with no other restraints than the family and friends can usually present, the patient must have his own way; and that way, to say the least of it, is not a good one, and often it is a very bad one. All the influences of home cannot prevent this, without exciting the very perversity they attempt to repress. When Mr. Pitt put king George III., under the care of Dr. Willis, in 1789, he was treated successfully, indeed, at his own home. But Dr. Willis discharged all the servants, attendants, lords and ministers, who had been about the king before. The family were all removed, the furniture taken away, and an entirely new corps of attendants placed about the royal patient, and the palace at Kew furnished so differently, and all the paraphernalia and regimen so thoroughly changed, that it had all the seeming of a removal from home ; for indeed the home was removed from him. The king recovered ; and so might our patients, without going from their own houses, if we could metamorphose those houses, and change the attendants, as Dr. Willis did for the king. This example is good for us, so far as it shows the necessity of placing the lunatic among strange men and objects.

Georget, one of the ablest of French writers on insanity, and himself long devoted to the observation and cure of this disease, says:* "Every physician who has been in the habit of attending to lunatics, has, without hesitation, advised the separation of such patients, in almost all cases, as the first condition and one of the principal means of cure. The insane ought to be removed from those objects, which first caused their alienation, or which might have kept it up or aggravated it; from relations and attendants whom they detest, whom they pretend to command, and whom they will not obey; from the inquisitive, who would irritate them by useless reasonings, or by ill-timed raillery. They ought to be removed from society, and placed in a habitation specially appropriated to this purpose, both for the safety of the public and for their own security."

Esquirol says: "The sensibility of the insane is perverted. They no longer have any relations with the external world, but those of a disordered and consequently painful nature. Every thing irritates them, distracts them, and excites their aversion. In constant opposition to all that surrounds them, they soon persuade themselves that persons are combined to injure them; and neither understanding what is said, nor being able to comprehend the reasonings, that are addressed to them, they misinterpret the most affectionate expressions, and the wisest counsels; they mistake the most candid, serious, and tender language, for insults, irony and provocations, and the most attentive kindness for contradictions. The regimen and the prohibitions, which are called for, by their situation, and to which their attendants wish to subject them, appear to them cruel persecutions."†

"The heart of the insane cherishes no feeling but mistrust; he is irritated to anger by every thing he sees, and he is so timid that he is troubled as soon as any one approaches him." "From mistrust, these patients soon pass to fear and hatred; and in these new moral situations, they repel their relations

* Dictionaire de Medicine. Art. *Folie.*

† Esquirol, Observations on the illusions of the Insane. Liddel's translation, quoted by Prichard.

and friends, and welcome strangers."* "With these moral dispositions, if left in the bosom of his family, the tender son, whose happiness used to consist in living near his mother, and in following his father's counsels, persuaded, that they wish to disgust him with his home, in order to drive him from it, falls into deepest despair, or escapes to destroy himself."*

"In mania or raving madness, the condition of the patient is such as to render the necessity of confinement obvious to common sense, both on account of the safety of the individual and that of his family, without adverting to the advantages resulting from such a measure in the promotion of recovery, and in the facility of applying remedies. In a great proportion of the cases of monomania, the propriety of adopting the same course is almost equally evident. The understanding is in this disease so disturbed, and the moral affections of the individual so perverted, that no alternative seems to be left."†

"It is now conceded by all, who are best acquainted with the management of the insane, that the first element in their moral treatment, is their removal from acquaintances and former associations. One prominent advantage in such removal, is the promotion of the second element of treatment, that of withdrawing the mind from its hallucinations, and attracting it into a new current of thought. New objects must be presented to the view, new incentives to the mind."‡

It is needless to quote more authorities upon this subject. They would be but repetition of the same opinion; for the observations of all have convinced them of the same truth. Within a few years, the States of New Hampshire, Connecticut, New Jersey, Pennsylvania, and Maryland, have appointed commissioners to investigate the condition of the insane poor in their limits. These commissioners have made reports,|| and all give melancholy proof of our position. They show, that of all the pauper lunatics, at their homes, or confined in jails, houses of correction, or poor-houses, none are cured, few alleviated, but most become confirmed in chronic

---

* Esquirol, Observations on the Insane: Liddel's translation, quoted by Prichard.

† Prichard. ‡ Earle, visit to 13 asylums. || Prison Dis. Soc. Reports.

insanity. The able and comprehensive Reports of the Prison Discipline Society confirm this opinion. And that society, true to its noble and benevolent purpose, has used its great influence to persuade sane men to give their insane brethren the earliest, and therefore, the best chance of relief in the institutions especially built and set apart for them.

It would seem almost useless to look abroad for any authority in this matter, when we have such abundant proof at home. Probably no inhabitant of the Western country, and very certainly no physician, has been so fortunate as not to have seen, in his own neighborhood, some lunatics whose insanity has grown old and confirmed, from neglect of proper and efficient means of relief. These being retained at their own homes, and in the bosom of their own families, their disorders have become chronic and perhaps hopelessly incurable. Such cases are familiar to us all; and who of us has seen a case of recovery under these domestic influences? Who of us has not seen instances of men changed, either by slow process, or by sudden transition, from mental soundness to mental derangement; from amiable gentleness to such irascibility of temper, as to make them almost or even quite intolerable in their families? And while the tender and affectionate relations and anxious friends are hoping, that another day or another month will remove the hallucination or wear the madness away, and restore the lunatic to his right mind and to their former confidence, that other day and other month pass by, and only leave the alienated patient a little and a little more confirmed in his madness. Their hope dwindles before the unrelieved, but strengthening disorder: despair succeeds to hope, and at last comes a bitter reconciliation to this afflicting dispensation, as men become reconciled, by habit, to any calamity, which they must endure. Of all these sorrowing friends that have hoped for this mental healing at home, how many have been gratified? And of all our profession, that have encouraged these distressed relatives to hope, in these circumstances, how many have found their promise fulfilled in the event?

The theory of the nature and the process of the disease, the testimony of all writers upon insanity, and of all those particularly devoted to its cure, and, more than all, our own observation prove, that home is not the place for the recovery of the lunatic, and that separation from familiar scenes and people is the first and a necessary step toward the accomplishment of this desirable object."*

---

* There are, of course, some exceptions to this general rule. There may be a few cases, which will not be benefitted by the removal from home. A very few may even be injured by being brought into the company of other lunatics.

When the disorder of the understanding is restricted to one or a few subjects, and the lunatic has his reason sound and clear on most matters, it is not so easy to determine the question of removal. Sometimes opposition to the will of a monomaniac, whose insanity is limited, may even disturb more of his sound faculties, and increase the insanity, or even create general mania.

If the monomaniac still retains his affections for, and his confidence in his family and relations, it may seem cruel to deprive him of their attentions. Nevertheless, if, notwithstanding his attachment to home, the subjects of his illusions are about his ordinary affairs, or haunts, or associates, it is still best to remove him.

Esquirol admits, that if the illusion of the insane relates to objects of indifference, and excites no strong emotions; if he has no aversions to his home and the persons with whom he lives, although confinement may be sometimes useful, it is not absolutely necessary."* "But if the patient, retaining a large portion of his intellect, has a strong attachment to his relatives, it is to be feared, that confinement might aggravate the disease."†

Some few melancholic patients may not be benefitted by a removal. Their dejection and gloom may be aggravated by such change, and particularly by the residence among similar patients.

Yet, after admitting these exceptions, the general rule of separation holds good; and though, like all rules of medical practice, they are neither mathematically exact, nor universally applicable, still the propriety of separating the lunatic from familiar persons and things, demands our first consideration, in every case; and we should be ever ready to do it; for almost all require it, and rarely is any one injured by it.

* Prichard. † Esquirol. Observations, quoted by Prichard.

It being then determined that the lunatic should be separated from his home and friends, the next question arises: Whither shall he be sent? Throughout the civilized world, and in Great Britain and America especially, this question has been very promptly and practically answered, with regard to a portion of lunatics at least. In all states, until within a few years, when a more enlightened and benevolent policy has in some been adopted, and in a few states, even now, imprisonment has been, and is the ready and efficient means of restraining the furiously mad. The uncontrollable and the violent, those who were dangerous to the public peace, such as committed assaults upon the persons or property of their neighbors, the bold maniacs, that kept fearful society in trembling awe of them, have been sent at once to the county jail, to the town work-house, or to some strong rooms especially prepared to hold them fast. This was a remedy for the public, but not for the patient. It restrained his hands, but it did not control the wanderings of his spirit, nor calm the madness of his feelings, nor confine the vagaries of his delusions. The utter worthlessness of this sort of confinement is most manifestly proved by the commissioners of New Hampshire, Massachusetts, Connecticut, New Jersey, Pennsylvania, and Maryland, in their very valuable reports upon the state and condition of the insane poor—very many of whom had been confined in their prisons and houses of correction for years—varying from one to forty, and not an instance of recovery is reported.* Not only no cures were obtained, but the report of Massachusetts says, with striking truth, "Were a system now devised whose express object it should be, to drive every victim of insanity beyond the limits of hope, it would scarcely be within the power of a perverse ingenuity, to suggest one more infallible than that which for so many years has been in practical operation among us."† So this plan is worse than useless.

---

* Prison Discipline Society Reports.

† State Lunatic Hospital Reports, p. 19.

TRAVEL—change of scene and occupation—placing the patient amid objects which may divert his thoughts, and change the course of his reflections; these are found beneficial in some recent cases of partial derangement.* But to say nothing of the great expense which such a course of treatment must require, we shall find but few, that would derive advantage from travel. The patient cannot be under the restraints that the cure of insanity requires, nor be kept from the external influences, that might aggravate the disease, nor be so well subjected to the suitable remedies.

It has been the practice with some, to send their insane patients or friends to board with some physician or other judicious person's family, remote from home, for the purpose of cure. For some cases, in which the patient retained his good temper, was easily managed, and required no restraint, this plan has been very happily successful. But most lunatics could neither be managed nor properly attended to in a private family. The needful and convenient means of governing and managing the insane, are not to be found in any private habitation. The master of such a house cannot have the authority, nor the servants the influence necessary for this purpose.† Georget says, that from an aversion to sending a lunatic to a public hospital, the friends sometimes send the insane to a house especially destined to receive a single patient, and there they surround him with strange attendants and servants. This is not only very expensive, but it fails of its object. For it is next to impossible to procure a perfect isolation; for the relations and friends are under strong temptation to visit the lunatic, and he very soon discovers that all this household and

---

* Prichard.

† "A private dwelling is ill adapted to the wants and requirements of such an unfortunate being. And even if it did contain all that is requisite, still there is little possibility that the patient could derive much benefit from persons who are neither acquainted with the proper system of treatment, nor if they were, could they possibly adopt it, and at the same time attend to any other business. A lunatic demands the whole time and attention of his guardians."—*Hill, on Insane Asylums, p.* 7.

preparations are for him, and then he is almost as little governed as if he were in his own family.*

This plan of placing the insane in private families, is too costly for most people, and is not sufficiently successful as a means of cure, to be advisable as a general rule for even those who can afford the expense.

Thus we see, that of these three modes of treating the insane, the first, imprisonment, is worse than useless—even exceedingly injurious. The second and third, travel and private boarding, are suited to but a small portion of cases; and are so expensive as to be beyond the reach of that small number who might be benefitted by them.

The only other remedy now proposed, is the HOSPITAL. In a preceding† article we demonstrated, that in the best asylums insanity was proved to be a very curable disease; as much so as fever, pneumonia, or dysentery. The power of the asylum is one grand triumph of modern science, over what has been hitherto supposed an unconquerable derangement; and in its latest improvement, it is the noblest institution of humanity.

In Kentucky, Governor Adair said, in 1821, that the old system of supporting lunatics had proved to be wholly inadequate to purpose of restoration of mental soundness.‡ This was a matter of commonest observation, and familiar to the eyes of all. In the establishment of the hospital there was nothing to be lost, but every thing to be gained. The earliest hospitals of Europe did not advance much. They were but a better sort of prisons. But Pinel's|| influence began a reform in these, which benevolent men elsewhere have carried

---

* Dict. De Medicine, Art. Folie.

† November Number, p. 449, etc.

‡ Message, 1821.

|| See that most interesting account of Pinel's unchaining the lunatics of the Bicetre, in 1792, in Browne, Hill, British and Foreign Medical Review, No. I. p, 286; Pris. Dis. Soc. Reports, and also North American Review, Vol. XIV. p. 103.

on from time to time, as light and love beamed upon them. These improvements, in the treatment of insanity, have been the most rapid within the last ten years. Pinel had no conception of the extent, to which his principles would ultimately grow, nor of the power which they were destined to acquire. When Governor Adair, in 1821, proposed to this state to establish a Lunatic Asylum in Kentucky, he said, "if only one out of twenty of those unfortunate beings, laboring under the most dreadful of all maladies, should be restored, will it not be a cause of gratulation to a humane and generous public?"* If to the eye of this benevolent magistrate, the recovery of one in twenty lunatics would justify the building and maintaining a hospital; and the probability of cure, amounting to only one-twentieth of a certainty, were reason sufficient for sending a patient from his home to the asylum, how would his noble heart have rejoiced to see that this very institution, which he recommended with such feeble hope, now cures eight times as many of all that are sent, and almost twelve times as many of the recent cases?† And still more, that in the more modern institutions, ten times as many are cured as he thought would warrant the abandonment of the old and the adoption of the new system.

In the United States, there are nineteen public and two private asylums for the insane. Four of the former are in the Western country—at New Orleans, Nashville, Tenn., Lexington, Ky., and Columbus, Ohio. All others are beyond the Alleghany mountains, and can be reached by us only through a long and tedious journey. We will now examine the various and particular merits and advantages of each of these institutions, so far as they concern us of the West. But before entering upon this examination, it is well to fix in our minds clearly, what we are to hope for, and what we may reasonably expect.

I. We hope for complete restoration of our patients to reason.

---

* Message. Rep. Journal. † See Report for 1841.

II. If this be not attainable, we hope for such amelioration of the disorder, that the patient may live peaceably and comfortably in his own family and among his own friends.

III. If, after all, he cannot be rendered safe to be at large, or at his house, then we desire that he have a comfortable home, where he will neither be exasperated nor neglected.*

IV. We desire, that the means used for these purposes, be of the gentlest and most judicious kind; that none of the severity of olden time be applied; but that all the tenderness and affectionate delicacy of home be used, in the new abode; that restraint be only so far applied as to prevent self-injury, or harm to others, and guidance sufficient to direct the wandering thoughts to a right channel.†

Judging by the results of the best asylums, we may reasonably expect the recovery of eighty or ninety out of every hundred, who have been insane less than one year; and the recovery of ten to twenty in a hundred of those, whose disorder has been of longer continuance. Or applying the doctrine of chances, there is eight to nine-tenths of a certainty, that any recent‡ case will recover, and one to two-tenths of a certainty, that any chronic case will be restored.

If any case, old or recent, be not curable, we may expect

---

* See Dr. Ray's Report on the Maine Asylum, for 1841.

† "It cannot be too widely made known, that in a properly constructed and well regulated asylum, the insane may be treated not only much more easily and effectually, but also *much more mildly* than at their own homes."—Report of the Lincoln, Eng. Asylum, for 1837, quoted by Hill.

‡ We use the word *recent* to express those who have been insane one year and under; and *old* or *chronic* for those insane over one year. In this sense are these terms used by most writers; and in all reports quoted in this article, except those of the Vermont asylum, which include in the recent class, only such as have been insane six months and under, and in the chronic class, those who have been disordered more than six months.

These terms, "recent," and "old," refer only to duration, and include all, within their respective limits of time, whatever may have been the cause of the malady. The numbers used here and hereafter to designate the probability of cure, refer only to the average of cases, arising from all sorts of causes. But under this average, there must be a great a great probability, that he will be so much improved that the maniac will cease to rage, the excitable will be quiet, and the distrest be comforted, so that they may live in their own families, and be tolerable in society.

We may confidently expect, that this restoration or improvement will be accomplished by such gentle means as would not shock the feelings of the tenderest relative.

Let us now see, in the first place, how far the several insane asylums of our country are accessible to the people of the West; and in the second place, how well they can fulfil our expectations of cure, amelioration, and gentle treatment of our patients.*

*The Insane Asylum at New Orleans* is simply a building in the yard of that noble institution, the Charity Hospital, of which it is a branch. It is built well, strong, and perhaps convenient; certainly it will answer the purpose of security. But there is such a small extent of grounds, so little room for exercise, so little preparation for labor, amusement, or other occupation, that surely they have not the usual, and what is elsewhere supposed, the necessary facilities for curing the in-

---

difference of probability, owing to difference of causes. Thus insanity produced by ill health is much more curable than that caused by masturbation.

Dr. Woodward, in his seventh report, gives the result of his observation of a thousand cases, and shows that of the whole number of cases, recent and old—

| | | | |
|---|---|---|---|
| Arising from ill health - - - | 63 $\frac{2}{3}$ | per cent | |
| Religious causes - - - - - | 58 | " | |
| Domestic afflictions - - - - | 56 $\frac{3}{4}$ | " | |
| Intemperance - - - - - | 49 $\frac{3}{4}$ | " | |
| Masturbation - - - - - | 23 $\frac{3}{4}$ | " | recovered. |

It would be interesting to know, whether the same relation of curability holds between these classes, in their recent and chronic states: whether the chance or probability of recovery diminishes with the age of the disorder, when produced by any one of these causes, more rapidly than when produced by any other.

* An account of these asylums was given, for another purpose, in this Journal, page 442, Vol. IV. No. VI., to which the reader is referred for a fuller notice of some of them, than is here given.

sane ; and we ought not to expect so much from them as from others. This asylum, however, has as yet hardly gone into operation, and we leave it to develope its powers, and to manifest its results ; by these shall it be judged hereafter. But, whatever may be its success, it is situated at the extreme South, and in New Orleans ; and therefore, as our lunatic patients must stay in a hospital for months or years, until they recover, this institution is only suited to the people of that latitude, who are accustomed to the diseases of that climate, and to the peculiar epidemics of that city.

*The Tennesee Lunatic Asylum* is richly endowed by the state, and can accommodate about a hundred patients. Farther than this we are not informed. We know nothing of its means and facilities for the management and cure of insanity, yet we believe the liberal grant from the government affords it means of great usefulness ; and we trust, that the reports will show a success corresponding to our expectations. It is intended to receive both the insane poor and pay patients.

*The Kentucky Lunatic Asylum* was established before the late discoveries had shown, how far this disease was controllable by attention, skill, and most faithful and tender watchfulness, in conjunction with variety of occupation. The original plan did not include a physician exclusively devoted to the institution, nor workshops, nor riding nor reading, nor a great variety of attendants. Nor was labor at first designed as one of the great means of improvement. A chaplain and religious worship was not then considered necessary for such an institution. A physician is engaged to visit the Asylum once a day; but his pay for this is so small, that he cannot neglect his general practice, to spend much time among the lunatics. A few attendants are employed, rather to guard and wait upon the patients, than to be their companions to guide their thoughts or control their feelings. Since the establishment of this asylum, the State has not altered the original plan, nor provided officers, and attendants, and means, according to the spirit of this improved age. But an effort is now making in the legislature to obtain such farther grants and privileges from the State, as will place this institution on as good a foundation as the best in the United States. When this shall be done, and a permanent resident physician, a chaplain, and a suitable corps of attendants shall be provided—when sufficient shops, lands, and all other means of labor abroad, and all facilities for occupation in the house, shall be obtained, then, we doubt not, this will take rank among the first institutions of the country, and be as desirable an abode for the lunatics of this and other western states, as any other asylum is for the insane of its region.

By the reports of 1839 and 1840, we find, that, counting the patients received in these two years, twenty-eight per cent of the recent cases, and thirteen per cent of the old cases were cured—counting the number discharged—forty-one per cent of the new cases, and thirteen per cent of the old cases were cured.

By the report for 1841,* we find that the success was greater

---

**Statement of the state of the Asylum for* 1841.

| | *Old cases.* | | | *Idiots and Epileptics* | | | *Recent cases.* | | | *Total.* |
|---|---|---|---|---|---|---|---|---|---|---|
| | Male. | Female. | Total. | Males. | Females. | Total. | Males. | Females. | Total. | |
| Remaining Jan. 1, 1841 | 49 | 53 | 102 | 16 | 19 | 35 | 2 | 3 | 5 | 142 |
| Admitted since | 26 | 12 | 38 | 6 | 4 | 10 | 18 | 6 | 24 | 72 |
| | 75 | 65 | 140 | 22 | 23 | 45 | 20 | 9 | 29 | 214 |
| Of whom have died | 11 | 5 | 16 | 6 | 3 | 9 | 2 | 1 | 3 | 28 |
| Discharged well | 5 | 5 | 10 | | | | 11 | 6 | 17 | 27 |
| Improved | 4 | 1 | 5 | | | | 2 | | 2 | 7 |
| Remaining Jan. 1, 1842 | 55 | 54 | 109 | 16 | 20 | 36 | 5 | 2 | 7 | 152 |

If the foregoing successful results of the operation are presented with the present resources, what happy effects could we anticipate, would the Legislature authorize the Managers to improve and enlarge the means for moral and physical treatment! We want every variety of occupation to divert the mind from its curious and various illusions.

during that year; for, counting the discharges, there were, of the old cases, twenty-five per cent cured, and twelve and a half per cent improved. And of the recent cases, there were seventy-seven per cent cured, and nine per cent improved. Counting the admissions, there were of the old cases twenty per cent cured and ten per cent improved; and of the recent cases there were sixty per cent cured and eight per cent improved.

In consequence of the laws of Kentucky allowing the quiet and the peaceable to be maintained at their own homes out of the public treasury, and of considerable general prejudice against hospital establishments, few, but the worst cases either of the pauper or the wealthier classes, are sent to the asylum.* These can have, not only much less chance for

---

Besides agriculture and horticulture, various mechanic operations should be employed. Christian worship, libraries, music, dancing, walking and riding, with complete bathing establishments, are unconditionally required for thorough success. Ornamental as well as useful improvement of the grounds and gardens, are considerations not to be forgotten, as important to render complete such an establishment, for the sick mind.

With comparative little expense, Kentucky can have a retreat for her insane, both a pride to the State and a blessing to a class of human beings whose condition should enlist the noblest of our feelings."—*Dr. Bush's letter to the Commissioners of the Lunatic Asylum, Jan.* 1*st*, 1842.

*To corroborate our assertion in a former article on this subject—page 476 of this volume—that "the most obstinate and incurable cases are selected out of all in the State to be sent to this hospital," we here add from Dr. Bush's letter:

"This asylum receives a very large proportion of congenital idiots, and hopeless cases of epileptics: all of which class of patients must die sooner or later, no rational hope existing to improve or even prolong a miserable existence. Such patients constitute, every year, by far the greater proportion of deaths reported. A patient, when received by the commissioners, is never discharged unless he or she be cured. So that many State patients, comprising idiots, epileptics, old cases of all the various forms of insanity, considered the scientific world over as incurable, must accumulate from year to year, and thus constitute a great proportion of the standing residents in the house, while in the very nature of things the annual lists of mortality have, in this fact, under the most favorable circumstances, an unavoidable increase."

recovery from insanity, but also a much greater chance of death, in consequence of their broken constitutions, and their liability to other diseases.

Nevertheless, with all those difficulties, the Kentucky asylum offers, even to these unpromising classes, sixty hundredths of a certainty of cure to a recent case, and twenty hundredths of a certainty of cure to an old case.

This asylum was primarily intended for the insane poor alone; but, for some years, it has received other patients from this and other States at the cost of $2,50 per week.

*The Ohio Lunatic Asylum* is exclusively a State institution, and admits—1st, the poor and the violently troublesome; 2d, others belonging to Ohio at the cost of $3 per week. This is an establishment of the first class for usefulness and benevolence. It is liberally provided with all means for the amusement and occupation of the patients—with a farm and garden—with shops, and horses, and vehicles—with books and papers, games and musical instruments. And the administration corresponds to the physical means. Dr. Awl, the superintendant, is a man singularly adapted by his skill and moral character, to direct such an establishment. His heart and his hands are in the cause of suffering humanity. The doors of his institution are thrown open to the poor and the distressed, and his work is carried on as a great moral enterprise, upon the principles of Christian charity, and according to the active and liberal spirit of this enlightened age.* His assistant physician, chaplain, ond other co-operators, are men and women of discretion and tenderness, and perform their duty of managing and encouraging the lunatics, with the kindest faithfulness. Their patients are treated as unfortunate friends—suffering, but not guilty—to be governed, but not punished.

All these means are brought to bear, with admirable effect, upon the disordered understanding and perverted feelings. The various sorts of labor abroad and in the house—the reli-

---

* Reports.

gious services—the reading—the parties for conversation, music and dancing, occupy them, so that they forget their illusions. The frantic have no time to rage, and their sanity creeps upon them. By these means, they have cured eighty-five per cent of the recent cases, and forty-one per cent of the old cases discharged; and therefore we may look for eighty-five hundredths of a certainty of cure of an average recent case, and forty-one hundredths of a certainty of a cure of a chronic case.

This institution accommodates each patient with a room, but is only open to the residents of Ohio. There are in that State thirteen hundred and sixty lunatics and idiots, of which four hundred and sixty-six are at public charge. If we deduct from this total number all the congenital idiots, the imbecile, the paralytic and epileptic, and those in the last stages of senile insanity, we shall find, without doubt, at least five hundred lunatics, who may be considered as fair subjects for the remedial measures of the asylum, and of whose restoration much hope may be entertained. The public guardians and the private friends of these unfortunates should not delay to send them at once to Columbus, and give them the earliest and best opportunity for recovery. That institution, in its charitable provisions, offers every inducement for the poor to come; and in its elegant arrangements and facilities, it invites those who are more accustomed to the comforts and refinements of life. And if the people of Ohio regard their true interest, and the good of their alienated citizens, they will send every hopeful lunatic at once to their asylum; and in that case it will not hold them. Its present accommodations will not admit more than a quarter of those who need its soothing and healing care. But the liberality of the State that began so noble a work, will enlarge it, so that it may fully answer its great purpose.

These are all the institutions of the west, and they cannot contain a fifth of the lunatics of the west who ought to be in them. All the other hospitals are beyond the mountains, and patients have often been carried from this valley to be healed in those eastern asylums. To which then of these shall we send our insane?

The nearest insane asylum which is open to us, is the *Pennsylvania Hospital* near Philadelphia. This is an entirely new institution, though a branch of the old hospital* of that city, from which it received the insane patients in 1841.

"This new asylum is under the superintendence of Dr. Thomas Kirkbride. It is indeed a splendid establishment: no expense that would contribute to the suitable accommodation of the patients, having been spared, in its erection. It has a large farm connected with it, of which forty-two acres are surrounded by a high stone wall. Under the care of its intelligent superintendant, this will undoubtedly be one of the best institutions in the country."† This will be managed on the right principles of kindness, watchfulness, and occupation. With all its facilities for governing and curing its inmates, with the high moral and scientific character of its head, and the discipline and discretion of its other officers and attendants, this institution makes high promise of good to its patients, and holds out great inducements for the lunatics of this and the other western States. Another year will tell, how well it can fulfil the great hope we have of it.

The cost for board and attendance is five dollars per week. Bond is required for the payment.

*The Friends' Asylum at Frankford* is six miles north of Philadelphia. Its farm, gardens, shops, houses, carriages, library, museum, games, billiard tables, chess and gammon, serve admirably their purpose of occupying the attentions and amusing the feelings of the insane. Religious services are also valuable auxiliaries to their other means of cure. The buildings are ample; they accommodate about seventy patients, and these are classified according to their sex, and to the kind and stage of the disease. This asylum is under

---

*The Pennsylvania Hospital is the oldest in the United States. It was established in 1752, and received lunatics as well as other patients. From 1752 to 1840, thirty-four per cent of lunatics were cured, including cases of mania a potu previous to 1823.

† Earle.

the immediate control of Dr. Pliny Earle, who has been long devoted to the interests of insanity and successful in treating it. The whole administration is of the mildest and most approved method of modern times. The officers and attendants are men and women of amiable tempers and bland deportment. No severity nor harshness are ever admitted there. Kind words, tender sympathies, and the sweet power of love, are their means for controlling the patients. No means are used but such as would be allowable, and even desirable, in private practice, in the midst of the most anxious relatives of the insane.

The reports state that, in twenty-four years, forty-two per cent of all the cases have been cured. But this asylum has improved with the age, and the later years have shown better success than this general average. In 1840, of eight who had been insane less thau three months, seven recovered, and one died. Of fourteen, who had been insane previously and recovered, and whose last attack was of less thrn three months standing; nine were cured, and five improved. Of twenty-one insane from three to twelve months, ten recovered, eight improved, one remained stationary, and two died. Of ten insane from one to two years, four were restored to health, three were improved, two unimproved, and one died. Of fifty-five* insane more than two years, three recovered, eight improved, four died, and forty were stationary.†

Taking these facts for data, for our hope of benefit in that institution, if our patient belong to the first class, he will have seven-eighths of a certainty of cure—if to the second class, (i. e. if insane less than three months, though not the first attack of the disease,) he may have sixty-four chances in a hundred for recovery; and if to the third class, his chance for recovery will be forty-seven in a hundred; if to the fourth class he may have forty-hundredths; and in the fifth class five hundredths of a certainty of restoration at Frankford.

---

* Only seven of these had been insane less than five years, and eight ess than ten years.

† Earle. Report for 1840.

In order to be admitted to this asylum, bond must be given by two persons—one of them at least to be resident of Philadelphia or its vicinity—to provide suitable clothing, to pay for all damages done by the patient, and to pay at least four dollars a week for board, medicine, and attendance; and to take the patient away on his discharge, or bury him at his death.

*The Bloomingdale Asylum* is on Manhattan island, six miles north from New York city, under the charge of Dr William Wilson. It receives one hundred and forty patients. During the twenty years of its operation, it has cured seventy-seven per cent. of its recent cases, and eleven per cent. of its old ones. In 1840, seventy-four per cent. of recent, and and thirty per cent. of old cases recovered, and nearly six per cent. died.*

Such success as this is evidence of good management. "The first endeavor is to classify the patients, and assign them to their proper attendants, who are each responsible for the personal cleanliness of their particular charge. Twenty attendants look after one hundred and thirty-five lunatics. These are expected to interest their patients, in their different amusements, accompany them in their walking about the grounds or in the neighborhood, take charge of them, when they ride out, and provide against escape or accident. All the patients who are in good health breakfast at seven, dine at one, and sup at six o'clock. In the interval between meals, they are generally employed in reading, walking, riding, or other amusement or occupation, and at proper seasons attend public worship."*

The directors "find that mild treatment, recreation, reading, and employment upon diverting objects, with amusements of various kinds, riding, walking, playing at games or on musical instruments, are much better calculated to remove gloomy impressions from the mind, than any course of harsh treatment."* We could add to these excellent means, labor,

* Report, 1840.

which has been most advantageously employed in other asylums.

This asylum is a branch of the New York City Hospital. It has very rich endowments, and receives ten thousand dollars a year from the state. Yet, on account of the very extensive and liberal accommodations and facilities for treating the patients, the cost for board, medicine, and attendance, is about $4 50 per week.

*The Connecticut Retreat for the Insane*, is at Hartford, in that most lovely valley of the Connecticut river. It is now under the care of Dr. Amariah Brigham, who has been long devoted to the philosophy and maladies of the mind. This asylum is furnished with all the apparatus for employment and amusement of the deranged inmates, which benevolence and skill have suggested. They have labor on land and in shops, riding in carriages, games of all sorts, books, newspaper and pamphlet literature, parties, music, dancing, and religious worship. Every thing is done by the watchful attention and faithful skill of the superintendent, and his fitting coadjutors, to relieve the distress, and to occupy the minds of the patients, to withdraw them from their illusions and to soothe the agony of their spirits.*

Beside Dr. Brigham, there are an assistant physician, a chaplain, thirteen attendants to take charge of seventy-nine patients, and four others, who have each the charge of one

* Report of Trustees.

The benevolent secretary of the Prison Discipline Society visited this hospital, and says: "We saw the apartments of the men; saw and saluted many of them as we passed through the halls, they appeared cheerful and courteous, and were glad to see us. The patients were in good condition, with respect to their persons and dress; the attendants polite, intelligent, and kind; the furniture, beds and bedding, all clean and sweet. The order of the establishment, thus far, was strongly in favor of improvement and progress toward a high standard. The superintendent showed much taste and skill, as well as urbanity, kindness, and controlling influence over mind, as we proceeded."—*Prison Discipline Society Report*, 1841.

lunatic, that may need particular attention, and ten other assistants to do the household work.

The whole management of this hospital is of the best kind; gentle, firm, watchful and affectionate; and it has been the most successful in the country: twenty-four per cent. of the old cases, and eighty-four per cent. of the recent cases have recovered, and six per cent. have died there. Supposing the present officers will do as well, (and we doubt not they will do better,) we may expect eighty-four hundredths of a certainty of cure for any average recent case, and twenty-four hundredths of a certainty of cure for old cases, that may be sent them. The whole cost of maintenance there is $4 per week.

The *Vermont Asylum for the Insane*, is also in that beautiful valley of the Connecticut, in Brattleboro, eighty miles north of Hartford. It is there surrounded by the richest scenery, composed of the grandeur of the mountains, and the loveliness of the highest cultivation and beauty of the meadows. Here nature alone might almost calm the maniac's frenzy, and cheer the drooping heart of the despondent.

This institution has ample accommodations for a hundred lunatics, and had ninety-five at the date of the last report, October, 1841. Each patient has a separate room in the night, and walks and mingles in halls common to the individuals of his class in the day. They are occupied with agricultural and mechanical labor—with public worship and reading—with riding, walking and games: and though watched with the most assiduous attention, they are never restrained by physical violence, nor overawed by harsh or threatening power. But it is made the duty of each officer and assistant to endeavor to secure the confidence and good will of the patients. For this purpose they treat them with the greatest kindness and forbearance.*

The zeal and discreet energy of the most faithful superintendent, Dr. William H. Rockwell, aided by a worthy body

* Report, 1841.

of associates, have been singularly successful in curing and improving the subjects of their charge. They have cured eighty-nine per cent. of all recent* cases that have been discharged, and twenty-eight per cent. of the old cases. If we suppose, that those remaining at the asylum under treatment to be as curable as those discharged, then we may look for a probability of cure, amounting to eighty-nine hundredths of a certainty for recent cases, and twenty-eight hundredths of a certainty for old cases.

*The McLean Asylum* for the Insane, at Charlestown, Mass., under the charge of Dr. Luther V. Bell, is one of the best endowed and best managed in the country. It has been many years in operation, and has been enabled to adopt every improvement, which its officers have discovered, or others have suggested. The high-toned benevolence of its trustees, the active observation of its physicians, have sought out all the means for the occupation and cure of the insane, that have been used in the hospitals of Europe or America.

The magnificent and convenient architectural arrangements of the buildings admit of the classification of the patients into at least a dozen different families, according to the developement and condition of the disorder, these have each their proper sitting, sleeping and dining apartments, &c.† The abundant means for the occupation of the alienated—the farm and elegant garden, filled with flowers and shrubs of every sort—the shops, and means for riding abroad—the library—the games of chess, gammon, draughts and cards—the musical instruments—the parties for sewing, dancing, &c.—the religious services, and, above all, the ever present and faithful companionship of the superintendent, the officers and attendants, all have the happiest influence over the vagaries of the deranged intellect, and the excitement or depression of the morbid feelings.

"The importance of securing the services of an elevated,

---

* Recent cases here include the insane six months and under.

† Report.

respectable, and cultivated class of persons for the responsible duty of attending upon the insane, was early recognized in this institution ; to obtain whcm no trouble or cost was to be spared. We have never been obliged to feel the want, which most writers upon insanity, and many institutions so feelingly deplore, of a proper kind of assistants. There are, in the interior of New England, a class of young men and women of respectable families, adequate education, and refined moral feeling, who are willing to devote themselves for a few years to this calling, under the encouragement which is offered them, of a fair pecaniary recompense ; and what is a still higher inducement, that of knowing, that their services are deemed of a highly respectable character. We never employ those in whom we would not place implicit confidence."* "The attendant will cheer the desponding, check the noisy or the petulant, turn the thoughts of those occupied in insane illusions, into a new channel ; walk, ride, and engage in amusements and employments with them."* Thus, none but persons of the sanest minds, the purest principles, and the most disciplined habits, the most amiable tempers and blandest manners, are allowed there to come in contact with the insane. The most perfect sanity is brought to bear upon insanity, even in the most casual associations. None of the harsh physical restraints, that once were used in asylums, are there. None of the stout and hardened warders to stand sentry ; no reviling taunt, nor chilling authority, are there ; but the mildest persuasion, the most affectionate watchfulness, and the kindest companionship make that the home of the distracted; they calm the frenzied, and cheer the disconsolate.

From its high reputation, this institution has received very many difficult and incurable cases ; and on account of its expensiveness, the means and patience of friends get exhausted, and many patients are taken away, before a full trial is made of the influence of the hospital over their disease ; and therefore more are discharged uncured, or merely improved, in proportion to thc restored, than would be otherwise. Yet, even

* Bell's Report, 1840.

with this deduction from the per centage of recoveries, this institution has been remarkably successful. Under the management of its present able and excellent superintendent, in the year 1838, the asylum cured every recent case that was discharged, except such as were taken away before full trial, and those who died.* In other years, they had not quite this degree of success, and, like other asylums, were obliged to discharge some voluntarily, whose cases resisted all their efforts at restoration.

The late reports of this hospital do not discriminate between the old and recent cases, and therefore we are unable to give precisely the results of these two classes, as we have done concerning others. Yet we find that fifty-nine per cent. of all that were discharged during the last four years, were cured; and of all under care, less than five per cent. died in each year.

For admission, application must be made to Dr. Bell, Charlestown, or to either of the trustees in Boston. When practicable, they require a description of the patient's disease, its early manifestation and progress; an account of his habits, age, social and domestic relations, and of the course pursued with him during his alienation. They require a certificate from a physician, that he is insane; and a written request from his nearest friend or relative, that he be admitted into the asylum, and lastly, an obligation, signed by two responsible persons, engaging to pay for clothing, and other things necessary for the health of the patient—for the board and wages of a special attendant, if necessary, and for board and medical attendance, $4 50 per week, and also to remove the patient, when discharged, or pay funeral charges, in case of death.*

There are asylums at Colombia, South Carolina, and at Milledgeville, Georgia, from which we have received no reports; we only know, that their states have made liberal grants to them. But whether their benefits are confined to

* Report, 1840.

those states, or whether they are open to us, and therefore available to the southern part of the Mississippi valley, we cannot tell. We hope to receive their reports and to learn that their usefulness is commensurate with their means.*

Beside these public hospitals, there are some private institutions worthy of our consideration.

Doctors S. and G. H. White have one at Hudson, New York, in which they treated eighty-four patients in 1840, and discharged forty-eight. Of these last, eighteen were recent cases, and twenty-seven chronic. Of the former, fourteen were cured, two improved, and two died; of the latter, seven were cured, fifteen improved, and three died—making seventy-six per cent. of cures of recent cases, and twenty-six per cent. of old cases.† This must be the ground of hope for our patients, if sent there. "Family worship is continued daily, and with beneficial effect on the patients. We have no information in regard to labor and amusements,"‡ nor of their other means of cure.

Dr. Cutter has a private asylum at Pepperel, Massachusetts, which is furnished with the usual means for labor and amusement, formed in good hospitals, and is conducted upon the best modern principles, and is one of the best private hospitals for the insane in the country.§

In this review of the American asylums, we see that, twelve are open to us of the West,|| and four only of these are within this valley. The others are far distant—the nearest, that at Philadelphia, is at least, three hundred miles from the most eastern point of our navigation at Pittsburgh.

To which, then, of these shall we send our insane? We

---

* The Georgia asylum was intended for one hundred and sixty patients, and receives both the poor and the rich.

§ Boston Med. and Surg. Journal.

|| The state asylums at Williamsburgh and Staunton, Va., at Worcester, Mass., at Augusta, Maine, at Baltimore, and those at New York and Boston, are all exclusively for the states or cities to which they respectively belong.

must judge them by their fruits. We learn from the foregoing facts, that the Kentucky asylum offers less probability of cure than other better endowed and more improved institutions; though we believe the legislature will put it in the power of its administration to make this as useful as any other. As it is now, if our patient's means permit a distant removal, and if they do not live in Ohio, they will do well to cross the mountatns. Yet, if this be impossible, the institution at Lexington is so much better than all other modes of treating insanity in Kentucky, that we ought not to hesitate for a moment to send our patients to it.

The Ohio asylum is all that could be expected or desired, except in extent; for it cannot contain a quarter of the insane of that state, and to all others it is closed. With such a means of cure within reach, it is a matter of astonishment, that there are not five hundred enjoying its advantages.*

All of the eight open institutions east of the Alleghanies, have physicians devoted exclusively to their inmates. All are managed on the modern principles of benevolence and occupation. All have religious worship, exercise, and amusement. All except those in New York employ labor; and we may feel assured, that in whichever of these we may place our patients, though far from their natural friends, they will find friendship, and tender attentions, and the faithful skill, which the present age has developed. Yet, if among these offering each so great a promise of good, we were to make a selection, we would prefer the McLean asylum, on account of the great and liberal and judicious preparations for the cure and the comfort of its residents. And especially for such, as have been accustomed to the refinements and luxuries of life, are the whole material and administration of this establishment suited. But if long residence for a term of years be wanted, or if economy be desired, the Brattleboro asylum is most favorable on account of the beauty of its location, and

---

* We are glad to see that there is an effort making, in the legislature of Ohio, at this moment, to enlarge this asylum.

its cheapness. The Hartford asylum has been exceedingly successful and satisfactory. As to the others, we leave their statistics and their history to speak for them, and these accounts are honorable to them and cheering to humanity.

We have been thus minute in describing these asylums, because for want of a definite knowledge of their merits, and of a conviction of the necessity of sending our patients to some one of them, most of our lunatics have been suffered to remain at home, with only domestic remedies for cure—the acute cases have become chronic—the curable have grown hopeless—and now we have a maniac and imbecile army of four thousand four hundred and fifty in the valley of the Mississippi.

No class of disorders so certainly yield to proper remedial measures, as insanity. And, on the other hand, none so certainly become incurable, if neglected. The recuperative powers of nature have less energy to heal this than any other disease; therefore, more than all others, this should not be left to heal itself. We should be as well prepared to treat, or to furnish means of treating our lunatic patients, as we should our fever patients. It would indeed be singular folly, for a physician to wait until he is called to a case of dysentery or bronchitis, before he determines upon the general principles of treatment; and having seen his cases, then get his books, for the first time, to learn the pathology of his diseases and the action of his remedies, and while he is reading his lessons, permit his acute cases to rush on to death, or to fast entrench themselves in the chronic state! If this be unfaithful preparation for the responsibilities, which we assume, as the advisers of the weak and the disordered, what shall we say to ourselves, if we are unprepared to advise our friends what course to pursue with a lunatic, and how they may save him, before all hope shall be extinguished?

Suppose a son of a family, that put their trust in us for counsel in sickness, should be taken delirious. He becomes irritable, violent, uncontrollable. He is suspicious of his best friends, and abusive of those, whom he before loved most.

His distressed parents, who had doated on him, and had rested every hope in him, know not what to do. In this agony, they send for us. We have been their medical guides, in all other derangements, and they rely upon us now, in their present grief, to tell them what to do. Shall we tell them, that we ourselves do not know what to do?—that insanity is mostly incurable?—that we can only bleed to bring down excitement, or give opium to quiet the nervous agitation? Shall we advise the restraint of chains to secure the family from being injured by him? Or confinement to prevent his running away? Or shall we not rather have our minds as well fortified for this emergency of mental disorder, as for the chance of meeting an attack of cholic or a fractured limb?

Having made our selection of an asylum as the means and interests of our patients dictate, we should not hesitate to send them to it, as early as possible after the attack of theii disorder. We must not be deceived by any fallacious mildness of the early manifestations of the lunacy; nor think that because the symptoms do not grow violent, it is safe to wait, for the disease may be fast fixing itself within, and becoming daily more difficult to be removed. The derangement, which at first is only one of function, gradually grows to be one of structure. A few days' or weeks' delay may carry the disorder beyond the point of curability. Where that point may be, cannot be determined beforehand; but it certainly is the safest to begin the remedial measures as early as possible. Dr. Woodward estimates, that the chance for recovery, if taken within the first three months after the attack, is double of that in cases which do not begin treatment until the ninth month. Dr. W.'s tables show, that of cases of one year or less duration, eighty-five per cent; of cases of from one to two years standing, fifty-seven per cent; of those of from two to five years continuance, thirty-four per cent; and of such as had been existing from five to ten years, eleven per cent were curable. Dr. Tuke gives seventy-nine per cent of cures of cases less than three months—forty-four per cent of those from three to twelve months, and twenty-five per cent of those of more than one year's standing, as curable. The reports of every hospital give a similar discrepancy between the curability of the recent, and that of the old cases, and manifestly prove, that we have every thing to gain for our patients, by early attention to their cure, and every thing to lose by delay.*

Having settled the question of removal, this should be done in all openness and sincerity. Deception should never be allowed toward the insane, for any purpose whatever. Dr. Allen says, "I consider it a point of the first importance, that truth should never be violated. If we begin by destroying confidence, we destroy the basis on which alone all moral good can be effected. It is quite a mistake to suppose, a system of deceit is necessary for the more quietly accomplishing their removal from home."† "I delicately and candidly tell them, that they are considered to be insane, that the disease has produced some change in their usual mode of thinking and feeling; that the object of the proposed visit is for their good."‡ "In this respect we should deal with them as we would with any reasonable person: we should tell them, that as they are deranged, another course is necessary, and it is for their benefit that we propose to remove them; and very few will object. But if they do object to the removal, we can inform them, that their going is a matter quite settled, and cannot possibly be altered."|| And even then they will submit with much better grace, than if we deceive them, and

---

* Such lunatics as do not show positive signs of incurability, and whose disorder offers any chance of cure, should be submitted to treatment at the earliest possible moment. We cannot commence too promptly the use of appropriate means. There is no doubt that insanity could be cured with much more ease, and much more frequently, if our remedial measures could be applied, at the very onset of the disease.---*Georget, Dict. de Medicine. Art. Folie.*

"The probability of recovery decreases in proportion to the length of time which may have elapsed between the period of attack and that of the removal."—*Hill, on Insane Asylums, p.* 9.

†On Classification, p. 29. ‡Ibid. ||Ibid.

pretend we are going to another place, while we are, in reality, going to an asylum.

The directors of these hospitals complain very much of this deception, which is frequently practised to induce patients to go to them. Vexed and disappointed to find themselves in a place of confinement, which they did not expect, they are excited, and resist the first efforts at cure; or they are downcast and sulky, and refuse to engage in the usual avocations for relief. But in the best asylums this deception is removed at once. Dr. Bell, of the McLean Asylum, says, "Our first care is to have the accompanying friends communicate to the individual, in our presence, if this has not been previously done, as it should be, where he is, that he is brought here as a deranged person, that his stay will depend upon the judgment of the physician as to his recovery; and he is made to understand, that the extent of his privileges will necessarily be dependent on his ability to comply with the rules, and to control himself." "However well he may appear, or however incoherent, this communication is substantially made to him, and no false representations are permitted to be made from this time henceforward in our intercourse with him."* Such is the practice of Dr. Woodward, and indeed of every faithful superintendent of any insane asylum.† Deception not only increases the difficulty of getting our patient to accompany us to his destination, but lessens the influence of the institution over him.‡

---

* Report, 1839.

† "No attendant or other person shall attempt to deceive or terrify any patient, or violate any promise made."—*Rules of the Lincoln Asylum.*

‡ It is a great error to suppose, that a lunatic cannot see facts and truth for himself, or that he can be cheated by false representations. In our pupillage, we saw a most affectionate mother offer a lunatic son a cup of valerian tea, saying, "Here's some good tea, my son." "Is it good?" said the maniac. "Yes." "But it is bitter." "No, my son, it is sweet." "Wont it hurt me?" "No, it will do you good." "Then, mother, if it is good, and sweet, and will do no hurt, let me see you drink half, and I will drink the rest." "No, my son, it is good for you,

From this examination we are led to the melancholy confession of the want of due provision for the comfort and the cure of the insane sufferers of the western country. In this broad and rich valley, from the lakes to the gulf of Mexico—from the Alleghanies to the Rocky Mountains, embracing a sane population of more than five millions, and lunatic population of more than four thousand, with no deficiency of wealth, skill, or benevolence, we have but four asylums for the insane. And these could not contain a tithe of all, who might be subjected to their influence, and not a fourth of those who could be benefited by them.

Even these hospitals, however excellent some of them may be, are intended primarily for the poor, and are therefore prepared and conducted, in a style necessarily more economical, than the richer classes would willingly pay for, or could enjoy, with advantage. And our pauper lunatics are sufficiently numerous to exclude all others. To accommodate this unfortunate class, we ought tc have public asylums in Indiana, Illinois, Missouri, Arkansas and Mississippi. And beside these we want then another asylum in the west, one of more elegant accommodations than ought to be expected in any State institution. The rich and the luxurious, the refined and the cultivated, are as liable to be bereft of their reason as their less fortunate brethren. There is a manifest propriety in providing for them in their sickness, buildings and comforts somewhat corresponding to what they enjoy in health. And we have no doubt, that an asylum of elegance and convenience, similar to the private institutions in the eastern States, if established near the great navigable thoroughfare of the western country, would soon be filled with patients, and do an immense service to society, and save many valuable citizens from irretrievable loss.

---

but not for me." "Then, if it is not good for you it is not good for me. You cannot cheat me to believe your nauseous medicine is nothing but good tea. I'll not touch a drop of it;" and the honest keen maniac spurned it from him. What a commentary upon the attempt to govern insanity by falsehood!

We want a hospital, in the west, to be planned and constructed, furnished and administered, according to the best ideas of the present age. From its very inception to its final operation, nothing should be overlooked or spared, that could directly or indirectly bear upon the comfort or the cure of the insane. Such an asylum should be situated near to the great thoroughfare of the West, near to the Ohio or the Mississippi river, for the convenient access of patients. "The situation chosen should be healthy. It should possess the advantage of a dry cultivated soil, and an ample supply of water; it should be so far in the country as to have an unpolluted atmosphere, a retired and peaceful neighborhood, and yet be so near a town, as to enjoy all the comforts and privileges, and intercourse, which can only be obtained in large communities."* Their daily wants can be better supplied, and the objects of interest can be much more easily and readily varied in the vicinity of a good market-town, than in the midst of a sparse population.

The location should not be a dead flat surface, nor in the midst of tame scenery. But "if the buildings be placed on the summit or the slope of a rising ground, the advantages are incalculable."† "To some the beauty of wood and water, hill and dale, convey grateful impressions." "To all a succession of new, and varied, and healthy impressions must be imparted."‡ There should be a large farm connected with the establishment, both for cultivation, walks, and other means of exercise, and also for the convenient distribution of the buildings. That acute observer of the means and arrangements of various institutions for the treatment of insanity, and their effects upon this disease—Louis Dwight—the Secretary of the Prison Discipline Society, says, there ought to be an acre of ground to a patient.|| Even twice this quantity would not be too much. For such an asylum as we propose, which would accommodate one hundred and fifty patients,

* Browne, p. 181. † Ibid. ‡ Ibid. ||Report, 1841, p. 16.

three hundred acres would be useful for the purposes of agricultural labor, and other exercises, and for the location of the houses, shops, &c., necessary for the establishment.

The architectural arrangement, and distribution of the buildings is of consequence both for the classification and for the facility of management of the patients. The usual, approved plan of the American asylums includes large centre buildings, and wings running from this to the right and left, and backward, proportioned to the wants of the institution. All the offices, dormitories, and other apartments, are under one roof. The asylum at Columbus, Ohio, one of the latest and best, is built upon this plan, and is copied from that excellent institution at Worcester, Mass.*

Esquirol prefers separate and low buildings. He says, that after having devoted ten years to reflection upon this subject; having personally examined all the French asylums, and the plans of many in other countries, and watched the effect of the one under his care, and the Saltpetriere, he has come to the conclusion that a lunatic asylum should not be in a city. But it should be on extensive grounds with an eastern exposure. The land should not be wet, yet well supplied with water. He prefers that there should be a centre building of one story, for the officers and their families. This should include the medical and receiving rooms, and apartments for sitting, eating, sleeping, &c. On the one side of this centre building, and running backward from it in a perpendicular direction, should be placed the houses for the patients. These should be separate structures, and sufficiently numerous for the classification of the patients according to the various kinds and periods of their malady. The maniacs which are furious, and those which are not mischievous; the melancholics who are noisy, and the quiet; the fatuitous, and the filthy; the epileptics, and those with other diseases, and the convalescent—these several classes should each have distinct habitations, entirely separated from each other. These dwellings

*For a full description of this, see former article on this subject.

should be of various styles of architecture, for monotony is wearisome to the lunatic as well as to the sane, and variety in this matter is one means of occupying the attention of the insane. They should be of only one story,* for the greater convenience of watching and serving the patients, and to prevent the danger of accidents incident to upper rooms and stairways, and for the readier access of the inmates to the yard. These houses should be built each with an interior quadrangular court, and include the sleeping-rooms, and the common parlors, eating-rooms, halls, offices, baths, for their respective classes of occupants. These houses will of course be built of material and in manner suited to the patients, that will occupy them. The violent will need strong rooms; the filthy will require paved floors; the suicidal will require padded walls, and the convalescent will enjoy light and genteel parlors as men in health.†

Browne says, "Modern establishments, instead of presenting an interminable succession of wards and corridors under one roof, generally consist of a number of separate houses, in which the patients are distributed according to their dispositions and the features and stage of their disease."‡

Dr. Allen, the proprietor and manager of a private asylum at High Beach, Norfolkshire, England, says—"I would have not only two establishments, but these sufficiently separated so as to prevent annoyance; and not only this separation, but I would have one to consist of a male and female part, sufficiently separated from each other. This arrangement I have, at my own establishments, which consist of Fair-Mead House, and Leopard's Hill Lodge, for males, and Springfield for females, with appendages and separate cottages. With two establishments, we can adopt a better and more complete method of classification."‖ And in all cases the habitations

---

*The French asylums are now principally built of one story."—*Browne, p.* 187.

†Esquirol, Des Maladies Mentales, Tom. II, p. 421.

‡Browne, p. 185.

‖On Classification, p. 3.

for "the noisy should be placed at a distance from the quiet patients, so as not to disturb them by their noise."*†

A plan of a very convenient asylum was devised by Dr. Lee and published in the Prison Discipline Society's reports for 1837. This consists of a centre building and many short lateral wings—all parallel with the front, but each retreating so far as to allow its central passage-way to open at each end into the open air.

To build such an asylum, with habitations, separated, isolated, and multiplied according to the kinds and stages of insanity in one hundred and fifty lunatics, would require at least three hundred acres of land. But this is not all. "A hospital building is but one item necessary for the successful management of the insane. In every possible case they should be employed. Riding, amusements, games, walks, and reading, are all useful, and the means for them all should be amply provided. But labor is the very best employment, and the only one that can be long continued without satiety. Provide fields, gardens, and workshops for labor, and a chapel for religious worship on the Sabbath, and you will show to the insane what you consider them capable of doing and enjoying; and they. in return, will show, by their industry, sobriety, and self-

---

* Browne, p. 185.

† "Pinel earnestly insists upon the necessity of classifying the insane; of separating those who would be injurious to each other, and putting together such as would be mutually beneficial in their cure. An asylum for lunatics should consist of several establishments, more or less separated from each other. There should be one fer each sex. There should be one for the excited, one for the tranquil, one for the convalescent, and one for those who have accidental diseases. It is well to have one division for the filthy, one for the demented, one for the furious, and noisy maniacs, and for such as cannot be controlled, whom we may send there for correction. More than all, it is important to separate the sexes, the convalescents, those who have been of bad morals or obscene in conversation, or licentious in their conduct. Each division should have a court planted with trees, and, as far as possible, a garden for the patients to walk in."—*Georget, Dict. de Medicine, Art. Folie.*

control, that they properly appreciate your confidence, and are grateful for your efforts to promote their happiness."* What these other means of occupation, labor and amusement are, we have described in our account of the best hospitals, in this and the preceding article on this subject.† A chapel, and means for religious exercises, are now found to be among the most important influences for the restoration of the reason. In no condition does the human mind approach its highest perfection so nearly as when in the act of worship of the Father of all Light and Truth.

Having provided liberally and faithfully the material of the asylum, it next behoves us to inquire,—Who should administer these and manage the insane? We have before spoken of the character of the officers and attendants as they are found in the most successful institutions in our country. In those, they are men and women of the healthiest minds, and of the highest mental and moral discipline, and so numerous, that one can give his whole attention to four or five patients; and if the case requires it, he may devote himself exclusively to one; for the grand secret, in the cure of insanity, is the power of sanity over it—the influence of the correct mind and heart over the disordered.

First, the asylum must have a physician for its superintendant, who shall give his entire and undivided attention and companionship to the patients. This is indispensable. Browne says—"The opinion was, and perhaps still is, prevalent, that if a building of suitable dimensions and security were provided, and if medical advisers occasionally saw the inmates, all was done for the insane that could be expected or that could be useful. Every day's experience shows, that these provisions are utterly inadequate to the end proposed—if that end be recovery, and not the confinement of the insane."‡

---

*Woodward, Hospital Report, p. 170.

†See this Vol., pp. 456—460. See also Reports of Ohio, Frankford, Bloomingdale, Hartford, Vermont, McLean, and Massachusetts hospitals.

‡Browne, on Insanity, p. 177.

But there must be a physician ever present, and he well qualified for his station. He must be a man of skill, self devotion and industry. He should be firm and courageous, yet of placid temper, and the gentlest manners. He must have a quick apprehension to discern the disposition and disorder of his patients, and tact to manage them. He must be benevolent towards man, and have a strong love for the particular branch of the profession which he assumes. "The basis of such a character must be dispositions truly Christian," and "there must exist a benevolent kindness, which shall be so deep and expansive, as not merely to feel sympathy for the lunatic because he is an alien to his kind, because he is visited with the heaviest and hardest affliction, which humanity can bear and live, but feel an interest in those unreal, artificial, and self-created miseries, with which the distracted spirit is oppressed. And this kindness will be as solicitous to alleviate suffering, where it is absurd, and the result of violence and perversity of temper, as where it flows from misfortune. There must be a benevolence which will be prepared to make the lunatic a companion and a friend."* The physician must associate with him on terms of reciprocal confidence, and mutual forbearance, of fellow feeling and rational counsel. He must forget, that an awful but not an impassable gulf of obliterated requirements, numbed or lethargic emotions, and darkened reason separates him from the maniac, but regard only the faculties they yet have in common, and make these the ground work of their intercourse. "There must be that benevolence which will imitate the mercy of Him, who in curing the broken and bewildered spirit of the demonom aniac, "took him by the hand and lifted him up." But this gentleness must be controlled. The merely benevolent physician can never be a good practitioner."† Such a one may be too indulgent, and while he is yielding to the tender impulses of his heart, and gratifying the temporary and capricious wants

---

*Browne, p. 179. †Ibid.

of his patients, he may be indulging vicious propensities, and encouraging and feeding those very delusions that are the cause of the derangement. "There must be mingled with this sentiment of benevolence that highly refined sense of duty, and that keen perception of right, which guides even kindness and affection in their ministrations, and which holds the balance as scrupulously in deciding on the moral rights of lunatics, as in determining the civil rights of our fellow-citi. zens."* The curator of the insane must have "that moral and physical courage and firmness, which confer calmness and decision in the midst of danger, and in dealing with the most furious and unlistening madness, and which imbues the whole character with a controlling influence, that, with mercy and justice, governs the turbulent, while it appears to guide them; and commands the most wild and ferocious, by the sternness, and, at the same time, by the serenity of its orders, showing neither timidity nor anger. The intellectual qualifications for such a trust are high and varied. They must comprehend a familiarity with the true and practical philosophy of the human mind, in order that its diseases may be understood and controlled, and a general acquaintance with the usages and workings of society; with the habits, pursuits, opinions and prejudices of different classes; with literature and science, so far as they contribute to the instruction, amusement, or happiness of these classes; with every thing, in short, that can be rendered influential in what may be called adult education, in the management or modification of character, in order that as great a number of moral means of cure, of restraining, persuading, and engaging the darkened and disordered mind, may be created as pessible. And finally, there must be as liberal a professional education as long study and observation can accomplish;"† so as to readily understand the causes of insanity, and the influence of the physiological state of the animal system over its duration or intensity, and the power

---

*Browne, p. 179. †Browne, pp. 178, 179.

of medicine, and of other moral and physical agents over either of these. "Such a physician is not a mere drug exhibiter,"* but he is a man of high principle and benevolence—of philosophy and practical wisdom. To such a man ought the whole establishment to be submitted for his care and faithful administration. This is the case with almost all the American, English, Prussian, Austrian, and many French asylums,† and is found to be best for the management of the insane.‡

Lastly, comes the provision of suitable stewards, officers, attendants, nurses, and servants. What should be the character and number of these, may be learned from the frequent notices and descriptions of them, which we have given in this and the former article,§ when speaking of the most successful assylums. Suffice it here to say—that they should have all the moral qualifications, and many of the mental accomplishments which we deem necessary for the superintending physician. This must, in no case, be overlooked, throughout the whole corps of attendants. From the head, to the lowest cook, sound minds, correct morals, and gentle manners must prevail,|| and if possible, all of these should be trained to their

---

* "To the man who conceives, that he can combat mania with the lancet and tartar emetic alone, or who believes that he can exorcise melancholia with a purge, it would certainly be unpardonable folly to commit the insane."—*Browne p.* 178.

† Esquirol.

‡ "It is absolutely necessary, that the physician be empowered with authority over every person and thing concerned in the peculiar service of the patients."—*Georget, Dict. de Medicine, Art. Folie.*

See also Esquirol, Des Maladies Mentales, Tom. II, p. 526. Des Maisons D' Alienes, §III.

§ Insanity and Insane Asylums, pp. 455, 458.

|| Consult, Brown on Insanity and Asylums for the Insane, Lecture V. Esquirol, Des. Maladies Mentales, Tom. II. p. 530. Also, Awl's, Earle's, Rockwell's, Bell's, Woodward's, Ray's, Lee's, and Prison Discipline Society's Reports, upon the character of officers and attendants.

Consult the same, and also Good, Prichard, Georget, and Abercrombie, upon the value of labor and amusement, and upon the influence of religious exercises in the treatment of Insanity; and likewise, Dr. Bush's late letter to the commissioners of the Kentucky Lunatic Asylum.

employment, before being entirely trusted with the care or service of the insane.*

With such provisions of lands, buildings, and other materials—with such officers and assistants—with religious service and light and laborious occupation of mind and body—a hospital might be of immense utility in this valley. And surely there is, within this wide reach of territory, and among these five millions of inhabitants, intelligence enough to appreciate such an institution; benevolence enough to desire it, and wealth sufficient to create it and put it into successful operation.†

---

See Dr. Lee's beau ideal of a perfect asylum. published iu the Eleventh Report of the Prison Discipline Society for 1836, and also his description of his own asylum at Charlestown, in the same.

See a beautiful account of Esquirol's private hospital at Ivry, by Dr. Andrew Combe, published in his treatise on "Physiology, applied to health and Education," Harpers' edition, p. 356.

* In many of the establishments in France, the keepers are required to have undergone a training previous to their appointment—Brown, p. 184. Several about to engage in this service, have resided for some time at York to observe the economy of the house and the management of the patients.—Tuke, Description of the York Retreat, p. 12.

† In addition to the authors and works herein referred to, Dr. Tuke's Description of the York Retreat, Miss Martinean's notice of the Hanwell Asylum, Conolly's, Spurzheim's, and Combe's works on Insanity, some articles in the American Journal of Medical Science, and Dr. Dunglison's pamphlets on the Insane Poor of Pennsylvania, may be well consulted, in corroboraiion of many of the statements and opinions given in this article.

---

NOTE.—We are happy to give testimony here to the great improvements of the Kentucky Asylum within the last year, under the faithful care of Dr. Bush. The Report for 1841, is more satisfactory than any that preceded it, and shows a greater success than in any former year. This account is corroborated by other and independent sources of information.

We are informed, that there is no doubt that the Legislature will grant to this asylum all the facilities that its warmest friends desire—a well paid physician, a sufficient corps of attendants, and lands and shops, for the occupation of the patients. When this shall be done, our own asylum will be second to none in the country.

